# Pathologic Characteristics of Somatotroph Pituitary Tumors—An Observational Single-Center Study

**DOI:** 10.3390/biomedicines11123315

**Published:** 2023-12-15

**Authors:** Agnieszka Tomasik, Maria Stelmachowska-Banaś, Maria Maksymowicz, Izabella Czajka-Oraniec, Dorota Raczkiewicz, Grzegorz Zieliński, Jacek Kunicki, Wojciech Zgliczyński

**Affiliations:** 1 Department of Endocrinology, Centre of Postgraduate Medical Education, 01-813 Warsaw, Poland; 2 Department of Cancer Pathomorphology, Maria Sklodowska-Curie National Research Institute of Oncology, 02-781 Warsaw, Poland; 3 Department of Medical Statistics, School of Public Health, Centre of Postgraduate Medical Education, 01-813 Warsaw, Poland; 4 Department of Neurosurgery, Military Institute of Medicine, 04-141 Warsaw, Poland; 5 Department of Neurosurgery, Maria Sklodowska-Curie National Research Institute of Oncology, 02-781 Warsaw, Poland

**Keywords:** acromegaly, somatotroph tumor, pathologic result, sparsely granulated tumor, densely granulated tumor, α-SU, Ki-67 index

## Abstract

The pathologic evaluation of a tumor tissue is an essential part of an acromegaly patient’s assessment. This study aimed to analyze the pathologic characteristics of pituitary tumors in patients with acromegaly. The demographic data, in addition to the hormonal, imaging, and pathologic results of 120 patients with acromegaly after pituitary surgery, were extracted from the Polish Acromegaly Registry. We compared sparsely and densely granulated tumors, GH(+), mixed GH(+)/PRL(+) and plurihormonal tumors, α-subunit-positive and α-subunit-negative tumors, and tumors of various Ki-67 indices in terms of the abovementioned features. Sparsely granulated tumors were more frequent in women than in men (*p* = 0.001) and in younger patients (*p* = 0.011), and they were larger (*p* < 0.001) compared to densely granulated tumors. Tumors with positive α-subunit were smaller (*p* = 0.013), showed extrasellar extension less often (*p* = 0.039), and were more often densely granulated (*p* < 0.001) compared to α-subunit-negative tumors. Patients with a higher Ki-67 index were younger (*p* < 0.001) and more often diagnosed with genetic syndromes (*p* = 0.02); they had higher GH concentrations (*p* = 0.007), larger tumors (*p* = 0.006), and cavernous sinus invasions more frequently (*p* = 0.022). Conclusions: The pathologic characteristics of somatotroph pituitary tumors are associated with patient’s age, sex, hormonal results, tumor size, and the degree of extrasellar expansion.

## 1. Introduction

Acromegaly is a rare endocrine disease, caused in most cases by a pituitary adenoma secreting growth hormone (GH). Patients usually present with typical appearance changes, headaches, excessive sweating, fatigue, and comorbidities such as hypertension, diabetes mellitus, osteoarthritis, or obstructive sleep apnea. However, acromegaly patients can differ a lot from each other in terms of disease course and treatment outcome. For instance, young patients more often present larger tumors, higher GH and insulin-like growth factor 1 (IGF-1) concentrations, and struggle with a persistent disease, whereas older patients may present mild disease with lower GH and IGF-1 concentrations and a good prognosis [1]. Such differences in tumors’ behavior affecting clinical course may be determined by certain tumor’s features. That is why pathologic evaluation of a tumor sample seems to be an important part of acromegaly patients’ evaluations. According to the new WHO classification of pituitary tumors from 2022, the pathologic assessment of somatotroph tumors should include immunostaining for anterior pituitary hormones, pituitary transcription factors, low-molecular-weight cytokeratins (LMWCs), proliferation markers (mitoses count, Ki-67 labeling index, and p53), and selected E-cadherin and somatostatin receptors (SSTRs) [2,3].

According to the density of GH-secreting granules in tumor cells and the presence of keratin aggregates called fibrous bodies on electron microscopy (EM), two types of tumors, sparsely granulated (SG) and densely granulated (DG) tumors, can be distinguished. DG tumors contain deeply eosinophilic tumor cells with a diffuse positivity for GH and a perinuclear staining pattern of LMWCs. In EM, they present a well-developed rough endoplasmic reticulum, abundant electron-dense GH-secretory granules of 300 to 600 nm, large Golgi complexes, and abundant perinuclear intermediate filaments. SG tumors are characterized by lightly eosinophilic tumor cells with fibrous bodies, focal positivity for GH, and diffuse small GH-secretory granules [4]. SG somatotroph tumors can be more aggressive and more invasive, which results in a poorer treatment outcome in acromegaly patients with SG tumors compared to those with DG tumors [5,6].

Alpha-subunit (α-SU) is one of two separate noncovalently bound subunits creating glycoprotein anterior pituitary hormones: TSH, LH, and FSH. It is identical in all three of the hormones listed above. Free α-SU may be detected in pituitary tumor samples using immunohistochemical (IHC) methods. Positive α-SU staining is more often detected in mixed GH(+)/PRL(+) tumors [7] and in DG tumors [8]. So far, little is known about the meaning of positive α-SU staining in acromegaly patients.

Antigen Kiel 67, also known as Ki-67, is a nuclear antigen associated with cellular proliferation, as it is present in all active stages of the cell cycle, i.e., the G1, S, G2, and M phases, but not in the G0 phase. It is associated with a tumor’s proliferation, invasiveness, and, consequently, prognosis [9,10,11]. It is usually detected using MIB-1 monoclonal antibodies (a mouse antibody intended for laboratory use in the qualitative identification of Ki-67 protein via IHC in formalin-fixed, paraffin-embedded human tissues).

Detailed knowledge on somatotroph pituitary tumor type and its pathologic characteristics allows us to understand some of the differences between acromegaly patients. In our study, we focused on associations between pathologic features of the tumor and the demographic, hormonal, and imaging characteristics of acromegaly patients. It is already known that clinical and pathologic characteristics may influence the treatment outcome in acromegaly [12,13,14]. In order to expand the possibility of predicting the treatment outcome in acromegaly and make it easier, we decided to concentrate on detailed patient characteristics, including pathologic features.

## 2. Materials and Methods

### 2.1. Patient Selection

One hundred and twenty patients with acromegaly treated in the Department of Endocrinology of the Centre of Postgraduate Medical Education in Bielanski Hospital in Warsaw, Poland were included in the present retrospective study. The inclusion criteria were a confirmed diagnosis of acromegaly according to the general criteria [12,15], at least one pituitary surgery regardless of surgical access, and an available pathologic result of the pituitary tumor sample. All patients included in the study were operated on by one of two neurosurgeons experienced in the pituitary field, either in the Department of Neurosurgery, Military Institute of Medicine, Warsaw, Poland or in the Department of Neurosurgery, Maria Sklodowska-Curie National Research Institute of Oncology, Warsaw, Poland. One hundred and sixteen patients were operated on using the transsphenoidal approach (96.67%), and four patients were operated on using the transcranial approach (3.33%). Eight patients (6.67%) were operated on more than once. In such cases, the pathological results from the last operation were analyzed. The data on demographics, hormonal results, clinical and imaging characteristics, as well as pathological results were extracted from the Polish Acromegaly Registry. The Polish Acromegaly Registry is a nationwide database covering data from patients with acromegaly treated in Poland who have given their informed consent to participate in the Registry. The Bioethics Committee of the Centre of Postgraduate Medical Education approved the project of creating the Polish Acromegaly Registry (approval 84/PB/2018). The Registry was created in 2017 and until 2022 it has gathered data from 445 patients treated in eleven Polish endocrinology centers. The extracted demographic data covered age, sex, and diagnostic delay, and the hormonal results included GH, IGF-1, and PRL concentrations at diagnosis of acromegaly and information on pituitary dysfunction. Clinical data comprised information on the diagnosis of a genetic syndrome with acromegaly and visual field defects. Imaging characteristics included maximal tumor diameter in mm and features of local invasion, i.e., extrasellar expansion, optic chiasm compression, and invasion of cavernous sinuses (no invasion, unilateral, or bilateral invasion). Pathologic results focused on IHC evaluation, including staining for anterior pituitary hormones and α-SU, proliferation index Ki-67, and tumor granulation pattern in EM.

The aim of this study was to characterize somatotroph tumors in the acromegaly cohort according to granulation pattern, IHC staining, and Ki-67 index and to compare various types of tumors in terms of clinical, hormonal, and imaging factors so that the pathologic results help to describe patients, which might be useful in personalized treatment planning. We separated SG and DG tumors; pure GH(+), mixed GH(+)/PRL(+), and plurihormonal tumors; α-SU-positive and α-SU-negative tumors; as well as tumors with Ki-67 < 1%, 1% ≥ Ki-67 < 3%, and Ki-67 ≥ 3% and correlated them with the clinical and imaging characteristics.

### 2.2. Pathological Evaluation

Pathological evaluation was performed by one pathologist experienced in pituitary pathology in the Department of Cancer Pathomorphology, Maria Sklodowska-Curie National Research Institute of Oncology, Warsaw, Poland.

Intraoperatively removed tumor fragments were fixed in 10% buffered formalin, embedded in paraffin blocks, cut into 4 µm sections, and routinely stained with hematoxylin and eosin. IHC staining was performed on paraffin sections using the EnVisionFlex/HRP Visualization System with DAB (3,3′-diaminobenzidine) as the chromogen (cat. no K8000, Dako/Agilent, Glostrup Kommune, Denmark). During the research period of about 20 years, antibodies from various companies were used, including Thermo Scientific (Waltham, MA, USA), Dako (Glostrup Kommune, Denmark), Invitrogen (Waltham, MA, USA), NeoMarkers (Fremont, CA, USA), Novocastra (Newcastle upon Tyne, UK), and BIO-RAD (Hercules, CA, USA). Staining procedures were performed in a Dako Autostainer Link48 (Dako, Denmark). The antibodies used for the majority of cases are as follows: antibodies against growth hormone (GH, cat. no. MS-1328-P1), prolactin (PRL, cat. no. MS-1362-P1), ACTH (cat. no. MS-452-P1) and β-TSH (cat. no. MS-1453-P1) from EPREDIA/Thermo Fisher Scientific Inc., Waltham, MA, USA; β-FSH (clone C10; cat. no. M3504), and β-LH (clone C93; cat. no. M3502) from Agilent/Dako, Denmark; glycoprotein α-subunit (cat. no. GTX75049, GeneTex Inc., Irvine, CA, USA); TP53 (DO-7; ready to use, cat. no. IR 616) and Ki-67 (MIB-1 clone, cat. no IR626) from Dako, Denmark); and anti-cytokeratin (Cam 5.2, cat. no. 06478425001) from Ventana Medical Systems, Inc., Tucson, AZ, USA.

EM examination was performed on 108 patients. In the remaining cases, no material for EM was obtained. Small tumor fragments were fixed in 2.5% glutaraldehyde and 1% osmium tetroxide, dehydrated in ethanol and propylene oxide, and then embedded in epoxy resin (Poly/Bed 812, cat. no 08791, Polysciences Inc., Hirschberg an der Bergstraße, Germany). Ultrathin sections were examined using a Philips CM120 BioTWIN transmission electron microscope (FEI Company, Hillsborough, OR, USA). Somatotroph tumors were classified on the basis of generally accepted histological and ultrastructural features, such as DG or SG adenomas.

### 2.3. Statistical Analysis

The data were analyzed using STATISTICA 13 software from StatSoft, Cracow, Poland. Absolute numbers (n) and percentages (%) of the occurrence of categories were estimated for categorical variables. Median (Me) with interquartile range (IQR) were estimated for numerical variables. The normal distribution of numerical variables was verified using Shapiro–Wilk’s test and based on a visual assessment of histograms.

The following statistical tests were used:

* Mann–Whitney’s U test to compare numerical clinical, imaging, or pathological variables between patients with DG and SG tumors, as well as between α-SU-positive and α-SU-negative tumors;
* Kruskal–Wallis H test to compare numerical variables between pure GH(+), mixed GH(+)/PRL(+), and plurihormonal tumors, as well as between 3 Ki-67 index intervals;
* Pearson’s chi-square test to compare categorical variables if all the expected counts were at least equal to 5;
* Fisher’s exact test to compare categorical variables if any expected counts were smaller than 5.

The significance level was assumed to be 0.05 in all the statistical tests.

Missing data were omitted in statistical analyses.

## 3. Results

### 3.1. Characteristic of the Study Group

Table 1 presents the demographic and clinical characteristic of 120 patients with acromegaly. Sixty-seven patients were females (55.83%). The median age of diagnosis was 44 years, and the median diagnostic delay was 5 years. The median fasting GH concentration was 11.7 µg/L, and the median IGF-1 concentration was 3.31 × the upper limits of normal (ULN). A total of 38 patients presented at least one pituitary axis dysfunction (31.67%), and 12 patients were diagnosed with one of the genetic syndromes associated with acromegaly (10.0%). The most frequent genetic syndrome was Multiple Endocrine Neoplasia Type 1 (MEN1) syndrome, diagnosed in six patients (5%).

Table 2 presents the imaging and pathomorphological characteristics of 120 patients with acromegaly. Ninety patients had macroadenoma at diagnosis (77.59%), and the median maximal tumor diameter was 19 mm. Sixty tumors had extrasellar expansion (51.72%), twenty-five compressed the optic chiasm (21.55%), and fifty-one invaded cavernous sinuses unilaterally or bilaterally (51.0%).

### 3.2. Comparison of Sparsely and Densely Granulated Tumors

Granulation pattern in EM was assessed in 108 patients. Forty-nine patients (45.37%) had SG tumors, and fifty-nine patients had DG tumors (54.63%).

Table 3 presents the comparison of clinical characteristics between SG and DG tumors. SG tumors occurred more frequently in women than in men. Patients with SG tumors were younger at diagnosis of acromegaly and presented thyrotropin deficiency at diagnosis of acromegaly more commonly compared to patients with DG tumors. We also observed a tendency towards more common visual field defects in SG tumors than in DG ones.

Table 4 presents the comparison of imaging and pathomorphological characteristics between SG and DG tumors. SG tumors were more common macroadenomas, were larger, presented extrasellar expansion and compressed the optic chiasm more commonly, showed more advanced cavernous sinuses invasion, stained positive for α-SU in IHC evaluation less often, and showed higher values of the Ki-67 index compared to DG tumors.

### 3.3. Comparison of Pure GH(+), GH(+)/PRL(+), and Plurihormonal Tumors

IHC for anterior pituitary hormones was assessed for 118 patients. Sixty-three tumors stained positive only for GH (53.39%), forty-six tumors stained positive for GH and PRL (38.98%), and nine tumors stained positive for at least three anterior pituitary hormones, including GH (7.63%). The most common plurihormonal tumors were GH(+)/PRL(+)/(LH(+) tumors (five patients). Three patients had GH(+)/PRL(+)/TSH(+) tumors and one had a GH(+)/PRL(+)/FSH(+) tumor.

Table 5 presents the comparison of clinical characteristics between the three groups of tumors listed above.

Patients with GH(+)/PRL(+) had a significantly higher PRL concentration and more common gonadotropin deficiency in comparison to patients with pure GH(+) and plurihormonal tumors.

Table 6 presents the comparison of imaging and pathomorphological characteristics between three groups of tumors: pure GH(+), GH(+)/PRL(+), and plurihormonal tumors. We did not find any statistical difference in imaging and pathomorphological characteristics between those groups of tumors.

### 3.4. Comparison of Tumors with Positive and Negative α-SU Staining

In total, 117 patients had α-SU staining assessed. A total of 55 tumors stained positive for α-SU (47.01%), and 62 tumors were negative (52.99%).

Table 7 presents the comparison of clinical characteristics between α-SU-positive and α-SU-negative tumors. We did not find any statistical difference in the clinical characteristics between those two groups of tumors.

Table 8 presents the comparison of imaging and pathomorphological characteristics between α-SU-positive and α-SU-negative tumors. Tumors with positive α-SU staining were smaller, more commonly DG tumors, and showed extrasellar expansion less commonly than α-SU-negative tumors. Moreover, we observed a tendency towards statistical significance for less common compression of the optic chiasm and cavernous sinuses invasion in α-SU-positive tumors compared to α-SU-negative tumors.

### 3.5. Comparison of Tumors with Various Values of Ki-67 Index

The Ki-67 index was assessed in 116 patients. A Ki-67 index < 1% was confirmed in 89 patients (76.72%), a Ki-67 index ≥ 1% and <3% in 16 patients (13.79%), and a Ki-67 index ≥ 3% in 11 patients (9.48%).

Table 9 presents a comparison of clinical characteristics between tumors with various Ki-67 index values. The patients with higher Ki-67 index values were younger, were younger at time of acromegaly diagnosis, had higher concentrations of fasting GH at diagnosis, and were more commonly diagnosed with a genetic syndrome associated with acromegaly. Among patients with a Ki-67 index ≥ 3%, 36.36% presented with one genetic syndrome associated with acromegaly (MEN1, McCune–Albright syndrome, or Carney syndrome), while in the group with a Ki-67 < 1%, only 6.74% did, *p* = 0.02. We also observed a tendency towards statistical significance for a shorter diagnostic delay and for more frequent thyrotropin deficiency in patients with higher Ki-67 values.

Table 10 presents a comparison of imaging and pathomorphological characteristics between tumors with various Ki-67 index values.

Higher Ki-67 values were associated with a larger tumor diameter and more frequent cavernous sinuses invasion.

All tumors with a Ki-67 index ≥ 3% were SG tumors, while 76.92% of tumors with a Ki-67-index ≥1% and <3% and only 35.37% of tumors with a Ki-67 <1% were SG tumors. We also observed a tendency towards statistical significance for macroadenomas in patients with higher Ki-67 values.

## 4. Discussion

The aim of the present study was to characterize somatotroph tumors according to pathologic evaluation and compare various types of tumors in terms of clinical, hormonal, and imaging factors in hope that the pathological results might help to describe patients and be useful in personalized treatment planning. This knowledge, i.e., how the factors above determine medical treatment outcome, may be useful in helping to select patients that would be resistant to standard first-line treatment. In such patients, the initiation of medical treatment using pegvisomant may be considered [16].

We showed that the pathologic results were associated with patients’ age, sex, hormonal results, tumor size, and the degree of extrasellar expansion. SG tumors occurred more frequently in women and in younger patients, and they were larger and more invasive compared to DG tumors. Positive α-SU staining was found more often in smaller, less invasive and DG tumors, whereas a higher Ki-67 index was observed in tumors of younger patients, in SG tumors, and was associated with higher GH concentrations; larger, more invasive tumors; and genetic syndromes with acromegaly more often.

The granulation pattern is important in the pathological assessment of somatotroph pituitary neuroendocrine tumors (PitNETs). It is usually performed by evaluating Cam5.2 keratin staining in IHC. However, in our study, EM was not used for that purpose. It is a less common method, which was recently supplanted by IHC. As far as evaluating the granulation pattern is concerned, these methods are equivalent. However, EM is advantageous in some situations requiring more accurate assessment, e.g., the characterization of unusual pituitary tumors or the distinction of mammosomatotroph from mixed somatotroph and lactotroph cell tumors [17].

The data available in the literature show different percentages of SG tumors in acromegaly; they vary from 20% to 75% [5,6,18,19,20,21]. In our cohort, it was 55.66%, with a slight predominance of SG tumors over DG tumors. However, it is worth mentioning that some authors differentiated between more than two groups of tumors according to the granulation pattern, i.e., apart from SG tumors and DG tumors, they also included intermediate tumors between SG and DG in their analysis. This makes the comparison of our results with others complicated. In our cohort, SG tumors occurred more frequently in younger patients compared to DG tumors, which complies with the previous literature [6,21,22,23,24]. The cited authors showed that diagnosis or operation age in patients with SG tumors ranged from 36 to 44 years, wherein the majority of them were indicated as being over 40 years and below 44 years. In our cohort, the median age at diagnosis in patients with SG tumors was 39 years. However, some results do not confirm that SG tumors occur more frequently in younger patients [19,20]. Similarly to Swanson et al. [20] and Larkin et al. [21], we showed that women presented with SG tumors more often than men. In our study, SG tumors were significantly larger than DG tumors reaching the median maximal diameter of 23 mm, which is in accordance with the results of most papers comparing SG with DG tumors [5,6,20,21,23,24,25]. In the publications mentioned above, the diameters of SG tumors fluctuated from 20 to 24 mm, whereas the diameters of DG tumors varied from 13 to 17 mm. DG tumors present higher hormonal activity and therefore more florid symptoms of hormonal excess, which leads to earlier diagnosis than in patients with SG tumors [3]. This explains our findings that larger SG tumors showed extrasellar expansion, compression of the optic chiasm, and cavernous sinuses invasion more often than DG tumors. We also showed that SG tumors were more likely to cause thyrotropin deficiency than DG tumors, whereas other pituitary axes were affected with similar frequency in both groups. The topic of pituitary deficiencies depending on the granulation pattern was not commonly mentioned; we found three papers mentioning it, but none of them confirm our findings. Larkin et al. showed more frequent gonadotropin deficiency in SG tumors compared to the DG tumors analyzed in macroadenoma cases; however, no differences in the frequency of thyrotropin deficiency were found between DG and SG tumors [21]. Sarkar et al. and Dehghani et al. did not find any statistically significant differences in terms of pituitary deficiencies between SG and DG tumors [5,18]. Comparison between SG and DG tumors in terms of the Ki-67 index showed higher Ki-67 values in SG tumors in our cohort, which was already mentioned in the previous literature [18,19,21]. In the current study, we compared SG and DG tumors in terms of α-SU staining, showing that DG tumors present positive α-SU in IHC evaluation significantly more often. The higher expression of α-SU in IHC in DG tumors was also described by Trouillas et al. [6,8]. Kiseljak-Vassiliades et al. assessed α-SU staining in both groups and obtained similar results to us [6]. Kontogeorgos et al. assessed α-SU production and release in DG and SG tumors in vitro. In their cohort, all DG tumors produced α-SU, and its production and release were significantly higher in DG than in SG tumors [26]. Except for the papers cited above, not much has been published so far regarding α-SU assessment in the IHC of somatotroph pituitary tumors. Thus, it is worth mentioning that our cohort is one of the largest published so far, as far as α-SU evaluation in the tumor sample is concerned.

In terms of the IHC evaluation of anterior pituitary hormones in tumor samples, we expected that plurihormonal tumors would have been larger and more invasive with more aggressive clinical presentation. However, our hypothesis has not been confirmed. The only statistically significant finding was the observation that GH(+)/PRL(+) tumors presented a higher PRL concentration and higher rate of gonadotropin deficiency, which complies with the common endocrine knowledge [4].

Regarding the proliferation index Ki-67, its meaning has already been widely analyzed in pituitary tumors. It has been shown that higher Ki-67 index values were correlated with the local invasiveness and clinical aggressiveness of the tumor [27,28,29]. Hasanov et al. showed that pituitary tumors with Ki-67 ≥ 3% presented more frequently as macroadenomas with cavernous sinus invasion, with higher surgery numbers and recurrence rates when compared to tumors with Ki-67 < 3% [27]. Stefanidis et al. confirmed positive correlation between the Ki-67 index and tumor size, extrasellar extension, cavernous sinus invasion, as well as recurrence rate [28]. Meanwhile, Sadeghipour et al. reported that the Ki-67 index was correlated with tumor invasiveness, but not with recurrence rate [29]. However, there is less data available on the correlation of the Ki-67 index, with tumors’ features and the clinical course of disease being in acromegaly. Most authors confirm that higher Ki-67 index values are associated with tumor invasiveness in acromegaly patients [30,31,32,33,34], which we have shown in our cohort as well. The above authors assessed extrasellar or suprasellar expansion and cavernous sinus invasion as features of invasiveness. Larger tumors were more often associated with higher Ki-67 values in our study group, but this association has not always been detected by other authors, even if they confirmed tumor invasiveness by higher Ki-67 [33,34]. Similarly to Mohseni et al. [35] and contrary to Fusco at al., Kasuki et al., and Huan et al. [32,33,34], we demonstrated that a patient’s age matters, and younger acromegaly patients present higher Ki-67 values. In Mohseni’s cohort, patients below 30 years presented a median Ki-67 index of 2.25%, while the median Ki-67 index in older patients was 1.0%. In our study, the median diagnosis age at Ki-67 > 3% was 29 years, and at lower Ki-67 indices, the diagnosis age stayed above 30 years. Hormonal results were also frequently analyzed in relation to the Ki-67 index. In our study, patients with a higher GH concentration presented higher Ki-67 values, which was also shown by Huan et al. [34]. What we find interesting is the fact that patients with genetic syndromes associated with acromegaly have tumors with higher Ki-67 index values, which may explain their more invasive character.

To sum up, our findings show that somatotroph tumors may be described by many pathologic features defining their biology. As we know from the literature, the cellular differentiation of pituitary tumors is important in tumor prognosis [36], and pathologic characteristics are of great predictive value [31,33,37,38]. Thus, the results of the current study are complementary with what we have published before [39] and may be helpful in personalized treatment planning in acromegaly patients.

## 5. Limitations and Strengths of the Study

Our study has some limitations that we list below. One of them is the retrospective design, as it caused missing data in some patients. Another one is the fact that patients operated on over almost twenty years were included, which resulted in changes in hormonal and pathologic assessment over that time and contributed to limited pathologic evaluation not including SSTR expression, protein p53, or E-cadherin. On the other hand, it is worth mentioning that our study includes one of the largest cohorts of patients among publications analyzing pathologic evaluation of the tumors in acromegaly. Only Swanson et al. gathered a larger cohort, but they assessed just the granulation pattern [20], and we have also analyzed IHC, including the Ki-67 index. What is more, our study is the first to compare α-SU-positive with α-SU-negative tumors in acromegaly.

## 6. Conclusions

In conclusion, our findings support an important role of the pathologic evaluation of somatotroph tumors as a part of acromegaly patient assessment. Younger patients present with more invasive tumors, which are more frequently SG tumors with a higher Ki-67 index. SG tumors occur more frequently in women and are larger and more invasive than DG tumors. Tumors with positive α-SU staining are smaller and less invasive than tumors with negative α-SU staining, and they are more often DG tumors. Tumors with higher Ki-67 index values are associated with higher GH concentrations, larger and more invasive tumors, and more frequent rates of genetic syndromes associated with acromegaly. Such pathological profiling could help clinicians to understand tumor behavior and predict the course of the disease. Thus, it might be a useful tool in selecting the most appropriate personalized treatment of acromegaly.

## Figures and Tables

**Table 1:** Demographic and clinical characteristics of 120 acromegaly patients.

Variable	Unit or Category	N	Statistics	Results
Age	years	120	Min–Max, Me (IQR)	23–74, 51 (44–62)
Age at diagnosis	years	120	Min–Max, Me (IQR)	15–69, 44 (35–55)
Sex	male	120	n (%)	53 (44.17)
	female			67 (55.83)
Diagnostic delay	years	113	Min–Max, Me (IQR)	1–29, 5 (3–10)
Fasting GH at diagnosis	µg/L	106	Min–Max, Me (IQR)	0.64–98.30, 11.70 (4.08–26.00)
IGF-1 at diagnosis	ng/mL	103	Min–Max, Me (IQR)	294–1600, 811 (618–1054)
	x ULN	103	Min–Max, Me (IQR)	1.18–6.35, 3.31 (2.55–4.34)
PRL at diagnosis	ng/mL	89	Min–Max, Me (IQR)	2–407, 14.5 (9.0–27.9)
Hyperprolactinemia at diagnosis	yes	89	n (%)	29 (32.58)
Pituitary axis dysfunction at diagnosis	at least one	120	n (%)	38 (31.67)
	FSH/LH			28 (23.33)
	ACTH			9 (7.50)
	TSH			9 (7.50)
Clinical diagnosis of genetic syndrome associated with acromegaly	at least one	120	n (%)	12 (10.00)
	MEN1			6 (5.00)
	FIPA			4 (3.33)
	McCune–Albright syndrome			2 (1.67)
Visual field defects	no	120	n (%)	102 (85.00)
	small			10 (8.33)
	quadrantanopia			8 (6.67)

N—number of patients for whom data were available. Results are presented as Min–Max, Me (IQR) for numerical variables, or n (%) for categorical variables. Me—median; IQR—interquartile range.

**Table 2:** Imaging and pathomorphological characteristics of 120 acromegaly patients.

Variable	Unit or Category	N	Statistics	Results
Tumor imaging in MRI	microadenoma	116	n (%)	26 (22.41)
	macroadenoma			90 (77.59)
Maximal tumor diameter	mm	102	Min–Max, Me (IQR)	5–51, 19 (11–25)
Extrasellar expansion	yes	116	n (%)	60 (51.72)
Compression of the optic chiasm	yes	116	n (%)	25 (21.55)
Degree of cavernous sinus invasion	no	100	n (%)	49 (49.00)
	unilateral			39 (39.00)
	bilateral			12 (12.00)
Granulation subtype of tumor	densely	108	n (%)	59 (54.63)
	sparsely			49 (45.37)
Immunohistochemical evaluation of tumor	pure GH(+)	118	n (%)	63 (53.39)
	GH(+)/PRL(+)			46 (38.98)
	plurihormonal			9 (7.63)
alpha-SU	(−)	117	n (%)	62 (52.99)
	(+)			55 (47.01)
Ki-67 Index	Ki67 < 1%	116	n (%)	89 (76.72)
	1% ≤ Ki67 < 3%			16 (13.79)
	Ki67 ≥ 3%			11 (9.48)

N—number of patients for whom data were available. Results are presented as Min–Max, Me (IQR) for numerical variables or n (%) for categorical variables. Me—median; IQR—interquartile range.

**Table 3:** Comparison of clinical characteristics between sparsely and densely granulated tumors.

Variable	Unit or Category	Statistics	Sparsely Granulated		Densely Granulated		*p*
			N	Results	N	Results	
Age	years	Me (IQR)	49	47 (38–59)	59	58 (45–64)	0.051
Age at diagnosis	years	Me (IQR)	49	39 (32–49)	59	46 (38–58)	0.011 *
Sex	male	n (%)	49	13 (26.53)	59	35 (59.32)	0.001 *
	female			36 (73.47)		24 (40.68)	
Diagnostic delay	years	Me (IQR)	46	5 (3–10)	55	6 (4–10)	0.201
Fasting GH at diagnosis	µg/L	Me (IQR)	43	12 (5.30–33.60)	56	10.45 (4.00–23.55)	0.274
IGF-1 at diagnosis	ng/mL	Me (IQR)	42	862 (632–1134)	52	806 (641–1020)	0.386
	x ULN	Me (IQR)	42	3.07 (2.53–4.30)	52	3.46 (2.80–4.36)	0.491
PRL at diagnosis	ng/mL	Me (IQR)	34	19.1 (9–34)	48	11.65 (9–24)	0.281
Hyperprolactinemia at diagnosis	yes	n (%)	34	14 (41.18)	48	13 (27.08)	0.181
Pituitary axis dysfunction at diagnosis	at least one	n (%)	49	17 (34.69)	59	19 (32.20)	0.785
	FSH/LH			12 (24.49)		15 (25.42)	0.911
	ACTH			3 (6.12)		5 (8.47)	0.642
	TSH			9 (18.37)		0 (0.00)	0.001 *
Clinical diagnosis of genetic syndrome associated with acromegaly	at least one	n (%)	49	6 (12.24)	59	5 (8.47)	0.519
Visual field defects	no	n (%)	49	38 (77.55)	59	54 (91.53)	0.060
	small			5 (10.20)		4 (6.78)	
	quadrantanopia			6 (12.24)		1 (1.69)	

N—number of patients for whom data were available. Results are presented as Min–Max, Me (IQR) for numerical variables or n (%) for categorical variables. Me—median; IQR—interquartile range. *p* for Mann–Whitney’s U test if numerical variables were compared between sparsely and densely granulated tumors, or *p* for chi-square or Fisher’s exact test if categorical variables were compared between those two tumors, *—statistical significance.

**Table 4:** Comparison of imaging and pathomorphological characteristics between sparsely and densely granulated tumors.

Variable	Unit or Category	Statistics	Sparsely Granulated		Densely Granulated		*p*
			N	Results	N	Results	
Tumor imaging in MRI	microadenoma	n (%)	48	4 (8.33)	57	21 (36.84)	0.001 *
	macroadenoma			44 (91.67)		36 (63.16)	
Maximal tumor diameter	mm	Me (IQR)	41	23 (20–34)	59	13 (9–20)	<0.001 *
Extrasellar expansion	yes	n (%)	48	36 (75.00)	57	20 (35.09)	<0.001 *
Compression of the optic chiasm	yes	n (%)	48	22 (45.83)	57	2 (3.51)	<0.001 *
Degree of cavernous sinus invasion	no	n (%)	41	12 (29.27)	49	30 (61.22)	0.002 *
	unilateral			19 (46.34)		17 (34.69)	
	bilateral			10 (24.39)		2 (4.08)	
Immunohistochemical evaluation of tumor	pure GH(+)	n (%)	49	30 (61.22)	57	29 (50.88)	0.562
	GH(+)/PRL(+)			17 (34.69)		24 (42.11)	
	plurihormonal			2 (4.08)		4 (7.02)	
alpha-SU	(−)	n (%)	48	36 (75.00)	57	20 (35.09)	<0.001 *
	(+)			12 (25.00)		37 (64.91)	
Ki-67 Index	Ki67 < 1%	n (%)	49	29 (59.18)	56	53 (94.64)	<0.001 *
	1 ≤ Ki67 < 3%			10 (20.41)		3 (5.36)	
	Ki67 ≥ 3%			10 (20.41)		0 (0.00)	

N—number of patients for whom data were available. Results are presented as Min–Max, Me (IQR) for numerical variables or n (%) for categorical variables. Me—median; IQR—interquartile range. *p* for Mann–Whitney’s U test if numerical variables were compared between sparsely and densely granulated tumors, or *p* for chi-square or Fisher’s exact test if categorical variables were compared between those two tumors, *—statistical significance.

**Table 5:** Comparison of clinical characteristics between pure GH(+), GH(+)/PRL(+), and plurihormonal tumors.

Variable	Unit or Category	Statistics	Pure GH(+)		GH(+) PRL(+)		Plurihormonal		*p*
			N	Results	N	Results	N	Results	
Age	years	Me (IQR)	63	56 (45–64)	46	48 (40–61)	9	48 (46–62)	0.231
Age at diagnosis	years	Me (IQR)	63	46 (36–58)	46	40 (33–50)	9	43 (36–51)	0.234
Sex	male	n (%)	63	26 (41.27)	46	23 (50.00)	9	4 (44.44)	0.656
	female			37 (58.73)		23 (50.00)		5 (55.56)	
Diagnostic delay	years	Me (IQR)	61	5 (3–10)	41	5 (3–10)	9	5 (1–5)	0.249
Fasting GH at diagnosis	µg/L	Me (IQR)	58	15.60 (4.10–33.60)	40	11.40 (4.38–20.55)	7	4.20 (2.80–14.50)	0.261
IGF-1 at diagnosis	ng/mL	Me (IQR)	56	861 (639–1102)	39	786 (617–990)	6	618 (420–917)	0.242
	x ULN	Me (IQR)	56	3.34 (2.76–4.63)	39	3.42 (2.44–4.20)	6	2.84 (1.59–3.46)	0.434
PRL at diagnosis	ng/mL	Me (IQR)	47	10.6 (6.3–20.2)	36	21.4 (11.2–39.4)	5	19.3 (14.8–102.0)	0.004 *
Hyperprolactinemia at diagnosis	yes	n (%)	47	11 (23.40)	36	15 (41.67)	5	3 (60.00)	0.087
Pituitary axis dysfunction at diagnosis	at least one	n (%)	63	15 (23.81)	46	18 (39.13)	9	5 (55.56)	0.070
	FSH/LH			9 (14.29)		17 (36.96)		2 (22.22)	0.022 *
	ACTH			4 (6.35)		3 (6.52)		2 (22.22)	0.234
	TSH			4 (6.35)		4 (8.70)		1 (11.11)	0.661
Clinical diagnosis of genetic syndrome associated with acromegaly	at least one	n (%)	63	6 (9.52)	46	5 (10.87)	9	1 (11.11)	0.999
Visual field defects	no	n (%)	63	54 (85.71)	46	39 (84.78)	9	7 (77.78)	0.880
	small			5 (7.94)		4 (8.70)		1 (11.11)	
	quadrantanopia			4 (6.35)		3 (6.52)		1 (11.11)	

N—number of patients for whom data were available. Results are presented as Min–Max, Me (IQR) for numerical variables or n (%) for categorical variables. Me—median; IQR—interquartile range. *p* for Kruskal–Wallis H test if numerical variables were compared between pure GH(+), GH(+) PRL(+), and plurihormonal tumors, or *p* for chi-square or Fisher’s exact test if categorical variables were compared between those three tumors, *—statistical significance.

**Table 6:** Comparison of imaging and pathomorphological characteristics between pure GH(+), GH(+)/PRL(+), and plurihormonal tumors.

Variable	Unit or Category	Statistics	Pure GH(+)		GH(+) PRL(+)		Plurihormonal		*p*
			N	Results	N	Results	N	Results	
Tumor imaging in MRI	microadenoma	n (%)	63	12 (19.05)	44	13 (29.55)	7	1 (14.29)	0.510
	macroadenoma			51 (80.95)		31 (70.45)		6 (85.71)	
Maximal tumor diameter	mm	Me (IQR)	55	20 (13–25)	38	15 (10–24)	7	20 (16–34)	0.229
Extrasellar expansion	yes	n (%)	63	32 (50.79)	44	21 (47.73)	7	5 (71.43)	0.570
Compression of the optic chiasm	yes	n (%)	63	14 (22.22)	44	9 (20.45)	7	2 (28.57)	0.818
Degree of cavernous sinus invasion	no	n (%)	53	25 (47.17)	39	22 (56.41)	6	2 (33.33)	0.584
	unilateral			21 (39.62)		12 (30.77)		4 (66.67)	
	bilateral			7 (13.21)		5 (12.82)		0 (0.00)	
Granulation subtype of tumor	densely	n (%)	59	29 (49.15)	41	24 (58.54)	6	4 (66.67)	0.557
	sparsely			30 (50.85)		17 (41.46)		2 (33.33)	
alpha-SU	(−)	n (%)	63	35 (55.56)	45	22 (48.89)	9	5 (55.56)	0.807
	(+)			28 (44.44)		23 (51.11)		4 (44.44)	
Ki-67 Index	Ki67 < 1%	n (%)	62	47 (75.81)	45	37 (82.22)	9	5 (55.56)	0.165
	1 ≤ Ki67 < 3%			9 (14.52)		6 (13.33)		1 (11.11)	
	Ki67 ≥ 3%			6 (9.68)		2 (4.44)		3 (33.33)	

N—number of patients for whom data were available. Results are presented as Min–Max, Me (IQR) for numerical variables or n (%) for categorical variables. Me—median; IQR—interquartile range. *p* for Kruskal–Wallis H test if numerical variables were compared between pure GH(+), GH(+) PRL(+), and plurihormonal tumors, or *p* for chi-square or Fisher’s exact test if categorical variables were compared between those three tumors.

**Table 7:** Comparison of clinical characteristics between α-SU-positive and α-SU-negative tumors.

Variable	Unit or Category	Statistics	alpha-SU (+)		alpha-SU (−)		*p*
			N	Results	N	Results	
Age	years	Me (IQR)	55	49 (42–61)	62	52 (45–64)	0.308
Age at diagnosis	years	Me (IQR)	55	45 (36–58)	62	43 (34–53)	0.236
Sex	male	n (%)	55	27 (49.09)	62	26 (41.94)	0.438
	female			28 (50.91)		36 (58.06)	
Diagnostic delay	years	Me (IQR)	53	5 (3–10)	57	5 (3–9)	0.131
Fasting GH at diagnosis	µg/L	Me (IQR)	51	11.70 (3.36–20.30)	54	11.90 (4.80–27.90)	0.211
IGF-1 at diagnosis	ng/mL	Me (IQR)	50	777 (625–981)	51	877 (614–1162)	0.113
	x ULN	Me (IQR)	50	3.34 (2.58–4.00)	51	3.42 (2.39–4.42)	0.750
PRL at diagnosis	ng/mL	Me (IQR)	48	14.7 (9.0–29.9)	40	12.9 (8.0–27.9)	0.750
Hyperprolactinemia at diagnosis	yes	n (%)	48	14 (29.17)	40	15 (37.50)	0.408
Pituitary axis dysfunction at diagnosis	at least one	n (%)	55	21 (38.18)	62	17 (27.42)	0.215
	FSH/LH			15 (27.27)		13 (20.97)	0.425
	ACTH			5 (9.09)		4 (6.45)	0.733
	TSH			5 (9.09)		4 (6.45)	0.733
Clinical diagnosis of genetic syndrome associated with acromegaly	at least one	n (%)	55	6 (10.91)	62	6 (9.68)	0.827
Visual field defects	no	n (%)	55	47 (85.45)	62	52 (83.87)	0.358
	small			6 (10.91)		4 (6.45)	
	quadrantanopia			2 (3.64)		6 (9.68)	

N—number of patients for whom data were available. Results are presented as Min–Max, Me (IQR) for numerical variables or n (%) for categorical variables. Me—median; IQR—interquartile range. *p* for Mann–Whitney’s U test if numerical variables were compared between α-SU-positive and α-SU-negative tumors, or *p* for chi-square or Fisher’s exact test if categorical variables were compared between those two tumors.

**Table 8:** Comparison of imaging and pathomorphological characteristics between α-SU-positive and α-SU-negative tumors.

Variable	Unit or Category	Statistics	alpha-SU (+)		alpha-SU (−)		*p*
			N	Results	N	Results	
Tumor imaging in MRI	microadenoma	n (%)	55	16 (29.09)	59	10 (16.95)	0.123
	macroadenoma			39 (70.91)		49 (83.05)	
Maximal tumor diameter	mm	Me (IQR)	51	16 (10–21)	49	20 (14–31)	0.013 *
Extrasellar expansion	yes	n (%)	55	22 (40.00)	59	36 (61.02)	0.039 *
Compression of the optic chiasm	yes	n (%)	55	8 (14.55)	59	17 (28.81)	0.066
Degree of cavernous sinus invasion	no	n (%)	47	29 (61.70)	51	20 (39.22)	0.081
	unilateral			14 (29.79)		23 (45.10)	
	bilateral			4 (8.51)		8 (15.69)	
Granulation subtype of tumor	densely	n (%)	49	37 (75.51)	56	20 (35.71)	<0.001 *
	sparsely			12 (24.49)		36 (64.29)	
Immunohistochemical evaluation of tumor	pure GH(+)	n (%)	55	28 (50.91)	62	35 (56.45)	0.807
	GH(+)/PRL(+)			23 (41.82)		22 (35.48)	
	plurihormonal			4 (7.27)		5 (8.06)	
Ki-67 Index	Ki67 < 1%	n (%)	54	45 (83.33)	61	43 (70.49)	0.234
	1 ≤ Ki67 < 3%			6 (11.11)		10 (16.39)	
	Ki67 ≥ 3%			3 (5.56)		8 (13.11)	

N—number of patients for whom data were available. Results are presented as Min–Max, Me (IQR) for numerical variables or n (%) for categorical variables. Me—median; IQR—interquartile range. *p* for Mann–Whitney’s U test if numerical variables were compared between α-SU-positive and α-SU-negative tumors, or *p* for chi-square or Fisher’s exact test if categorical variables were compared between those two tumors, *—statistical significance.

**Table 9:** Comparison of clinical characteristics between tumors with various Ki-67 index values.

Variable	Unit or Category	Statistics	Ki-67 < 1%		1% ≤ Ki-67 < 3%		Ki-67 ≥ 3%		*p*
			N	Results	N	Results	N	Results	
Age	years	Me (IQR)	89	55 (45–64)	16	47 (41–57)	11	39 (31–46)	0.001 *
Age at diagnosis	years	Me (IQR)	89	46 (38–58)	16	38 (33–47)	11	27 (22–39)	<0.001 *
Sex	male	n (%)	89	44 (49.44)	16	7 (43.75)	11	2 (18.18)	0.159
	female			45 (50.56)		9 (56.25)		9 (81.82)	
Diagnostic delay	years	Me (IQR)	84	5 (3–10)	15	4 (2–12)	10	3 (1–7)	0.096
Fasting GH at diagnosis	µg/L	Me (IQR)	82	9.84 (4.07–19.40)	13	20.30 (10.80–34.00)	8	53.00 (19.73–78.45)	0.007 *
IGF-1 at diagnosis	ng/mL	Me (IQR)	80	804 (616–1049)	13	940 (760–1075)	7	811 (621–1260)	0.549
	x ULN	Me (IQR)	80	3.42 (2.53–4.44)	13	3.45 (2.80–3.90)	7	2.75 (2.73–3.42)	0.572
PRL at diagnosis	ng/mL	Me (IQR)	71	12.1 (8.5–27.7)	9	20.2 (13.7–29.0)	6	21.7 (4.5–37.8)	0.559
Hyperprolactinemia at diagnosis	yes	n (%)	71	20 (28.17)	9	5 (55.56)	6	3 (50.00)	0.127
Pituitary axis dysfunction at diagnosis	at least one	n (%)	89	26 (29.21)	16	6 (37.50)	11	6 (54.55)	0.233
	FSH/LH			20 (22.47)		5 (31.25)		3 (27.27)	0.696
	ACTH			7 (7.87)		0 (0.00)		2 (18.18)	0.227
	TSH			5 (5.62)		1 (6.25)		3 (27.27)	0.069
Clinical diagnosis of genetic syndrome associated with acromegaly	at least one	n (%)	89	6 (6.74)	16	1 (6.25)	11	4 (36.36)	0.020 *
Visual field defects	no	n (%)	89	77 (86.52)	16	13 (81.25)	11	8 (72.73)	0.372
	small			7 (7.87)		2 (12.50)		1 (9.09)	
	quadrantanopia			5 (5.62)		1 (6.25)		2 (18.18)	

N—number of patients for whom data were available. Results are presented as Min–Max, Me (IQR) for numerical variables or n (%) for categorical variables. Me—median; IQR—interquartile range. *p* for Kruskal–Wallis H test if numerical variables were compared between tumors with three intervals of Ki-67 index, or *p* for chi-square or Fisher’s exact test if categorical variables were compared between those three tumors, *—statistical significance.

**Table 10:** Comparison of imaging and pathomorphological characteristics between tumors with various Ki-67 index values.

Variable	Unit or Category	Statistics	Ki-67 < 1		1 ≤ Ki-67 < 3		Ki-67 ≥ 3		*p*
			N	Results	N	Results	N	Results	
Tumor imaging in MRI	microadenoma	n (%)	86	24 (27.91)	15	2 (13.33)	11	0 (0.00)	0.076
	macroadenoma			62 (72.09)		13 (86.67)		11 (100.00)	
Maximal tumor diameter	mm	Me (IQR)	80	17 (10–25)	11	20 (16–21)	8	32 (26–36)	0.006 *
Extrasellar expansion	yes	n (%)	86	39 (45.35)	15	9 (60.00)	11	9 (81.82)	0.054
Compression of the optic chiasm	yes	n (%)	86	16 (18.60)	15	4 (26.67)	11	5 (45.45)	0.107
Degree of cavernous sinus invasion	no	n (%)	75	43 (57.33)	12	4 (33.33)	9	1 (11.11)	0.022 *
	unilateral			24 (32.00)		5 (41.67)		7 (77.78)	
	bilateral			8 (10.67)		3 (25.00)		1 (11.11)	
Granulation subtype of tumor	densely	n (%)	82	53 (64.63)	13	3 (23.08)	10	0 (0.00)	<0.001 *
	sparsely			29 (35.37)		10 (76.92)		10 (100.00)	
Immunohistochemical evaluation of tumor	pure GH(+)	n (%)	89	47 (52.81)	16	9 (56.25)	11	6 (54.55)	0.165
	GH(+)/PRL(+)			37 (41.57)		6 (37.50)		2 (18.18)	
	plurihormonal			5 (5.62)		1 (6.25)		3 (27.27)	
alpha-SU	(−)	n (%)	88	43 (48.86)	16	10 (62.50)	11	8 (72.73)	0.234
	(+)			45 (51.14)		6 (37.50)		3 (27.27)	

N—number of patients for whom data were available. Results are presented as Min–Max, Me (IQR) for numerical variables or n (%) for categorical variables. Me—median; IQR—interquartile range. *p* for Kruskal–Wallis H test if numerical variables were compared between tumors with three intervals of Ki-67 index, or *p* for chi-square or Fisher’s exact test if categorical variables were compared between those three tumors, *—statistical significance.

## Data Availability

The datasets presented in this article are not readily available because the dataset comes from the Polish Acromegaly Registry. The privacy policy and personal data protection rules assume only result sharing and not dataset sharing. Requests to access the datasets should be directed to Agnieszka Tomasik, agnieszka@tomasik.pl.

## Abbreviations

List (in the order they appear in the text).

GH	growth hormone
IGF-1	insulin-like growth factor 1
WHO	World Health Organization
LMWCs	low-molecular-weight cytokeratins
SSTR	somatostatin receptor
EM	electron microscopy
SG	sparsely granulated
DG	densely granulated
α-SU	α-subunit
TSH	thyrotropin-secreting hormone
LH	luteinizing hormone
FSH	follicle-stimulating hormone
IHC	immunohistochemistry
PRL	prolactin
Mm	millimeters
ACTH	adrenocorticotropic hormone
Me	median
IQR	interquartile range
ULN	upper limits of normal
MEN 1	Multiple Endocrine Neoplasia Type 1
FIPA	Familial Isolated Pituitary Adenomas
